# Proteomic Analysis of Aorta and Protective Effects of Grape Seed Procyanidin B2 in db/db Mice Reveal a Critical Role of Milk Fat Globule Epidermal Growth Factor-8 in Diabetic Arterial Damage

**DOI:** 10.1371/journal.pone.0052541

**Published:** 2012-12-21

**Authors:** Fei Yu, Bao-ying Li, Xiao-li Li, Qian Cai, Zhen Zhang, Mei Cheng, Mei Yin, Jun-fu Wang, Jian-hua Zhang, Wei-da Lu, Rui-hai Zhou, Hai-qing Gao

**Affiliations:** 1 Key Laboratory of Cardiovascular Proteomics of Shandong Province, Department of Geriatrics, Qi-Lu Hospital of Shandong University, Jinan, China; 2 Institute of Basic Science, Medical Science Academy of Shandong, Jinan, China; 3 Division of Cardiology, University of North Carolina at Chapel Hill, Chapel Hill, North Carolina, United States of America; Consiglio Nazionale delle Ricerche, Italy

## Abstract

**Background:**

Atherosclerosis is one of the major complications of type 2 diabetic patients (T2DM), leading to morbidity and mortality. Grape seed procyanidin B2 (GSPB2) has demonstrated protective effect against atherosclerosis, which is believed to be, at least in part, a result of its antioxidative effects. The aim of this study is to identify the target protein of GSPB2 responsible for the protective effect against atherosclerosis in patients with DM.

**Methods and Results:**

GSPB2 (30 mg/kg body weight/day) were administrated to db/db mice for 10 weeks. Proteomics of the aorta extracts by iTRAQ analysis was obtained from db/db mice. The results showed that expression of 557 proteins were either up- or down-regulated in the aorta of diabetic mice. Among those proteins, 139 proteins were normalized by GSPB2 to the levels comparable to those in control mice. Among the proteins regulated by GSPB2, the milk fat globule epidermal growth factor-8 (MFG-E8) was found to be increased in serum level in T2DM patients; the serum level of MFG-E8 was positively correlated with carotid-femoral pulse wave velocity (CF-PWV). Inhibition of MFG-E8 by RNA interference significantly suppressed whereas exogenous recombinant MFG-E8 administration exacerbated atherogenesis the db/db mice. To gain more insights into the mechanism of action of MFG-E8, we investigated the effects of MFG-E8 on the signal pathway involving the extracellular signal-regulated kinase (ERK) and monocyte chemoattractant protein-1 (MCP-1). Treatment with recombinant MFG-E8 led to increased whereas inhibition of MFG-E8 to decreased expression of MCP-1 and phosphorylation of ERK1/2.

**Conclusion:**

Our data suggests that MFG-E8 plays an important role in atherogenesis in diabetes through both ERK and MCP-1 signaling pathways. GSPB2, a well-studied antioxidant, significantly inhibited the arterial wall changes favoring atherogenesis in db/db mice by down-regulating MFG-E8 expression in aorta and its serum level. Measuring MFG-E8 serum level could be a useful clinical surrogate prognosticating atherogenesis in DM patients.

## Introduction

Vascular complications are the major cause of morbidity and mortality in patients with T2DM [1]. The hallmarks of macrovascular damage are accelerated atherosclerosis and remodeling of large arteries characterized by thickening and stiffening of the arterial wall [2], [3]. Thus far, there is no cure available for T2DM and its cardiovascular complications. Therefore, elucidation of the underlying molecular mechanism of atherosclerosis associated with T2DM and identification of the target protein critically involved in this process could lead to a more specific strategy in fighting against the vascular complications of T2DM.

The induction of oxidative stress, especially the reactive oxygen species (ROS) plays a critical role in the development of diabetic complications [4], [5]. Procyanidins are a complex family of polyphenol polymers widely existing in natural products such as grape wines, fruits and vegetables [6], [7]. Grape seed proanthocyanidin extracts (GSPE) derived from grape seeds. Dimeric procyanidin B2 is one of the main components of GSPE, composed of two molecules of the flavan-3-ol (-)-epicatechin linked by a 4b→8 bonds. Studies including ours have shown that procyanidin B2 has anti-inflammation, anti-tumor and cardiovascular protective properties, which are believed at least in part, to result from the antioxidative effects [8]–[12]. Our previous data showed that GSPB2 could prevent AGEs-induced ROS generation, inhibit the human umbilical vein endothelial cell (HUVEC) apoptosis, AGE-induced proliferation and migration of human aortic smooth muscle cells (HASMCs) [13]–[15]. These data suggest that GSPB2 could exert protective effect on the development of atherosclerosis in T2DM.

In an effort to understand the underlying molecular mechanisms and identify the potential target protein molecule in atherogenesis in T2DM, we resorted to proteomic comparative analyses of the aorta using db/db diabetic mice. db/db mice are well-established animal model to study T2DM complications. The quantitative proteomics using isobaric tags for relative and absolute quantification (iTRAQ) technique enables the detection and quantitation of proteins, including the hydrophobicity and high molecular masses of proteins. We found that expression of 557 proteins was altered in the aorta of db/db mice and 139 proteins among them including MFG-E8 were normalized by GSPB2 to the levels comparable to those in control mice. Our data suggest that MFG-E8 is a target protein of GSPB2 and plays an important role in atherogenesis in T2DM.

## Materials and Methods

Detailed materials and methods are described in Materials and Methods S1.

### Materials

GSPB2 (more than 90% pure, Lot No: 20100915) was purchased from Jianfeng Inc (Tianjin, China). Anti-mouse MFG-E8 antibody was purchased from Abcam (Cambridge, USA). Anti-human MFG-E8 antibodies and recombinant human MFG-E8 were purchased from R&D Systems (Minneapolis, USA). Anti-human MFG-E8 polyclonal antibody was purchased from Santa Cruz Biotechnology (Santa Cruz, USA). The ELISA kit of MCP-1 was purchased from eBioscience (Ssn Diego, USA). 8-plex isobaric tags for relative and absolute quantification (iTRAQ) protein labeling kit/reagents were purchased from Applied Biosystems (CA, USA). All other reagents used were standard commercial high-purity materials.

### Animals

Male C57BLKS/J db/db and db/m mice (n  = 24, 7 weeks old) were purchased from Model Animal Research Center of Nanjing University (Jiangsu, China). They were housed in standard animal cages and received laboratory pellet chow and tap water ad libitum in a constant environment (room temperature 20–22°C, room humidity 40–60%) with a 12-h light, 12-h dark cycle. The mice were acclimated in the animal housing facility for one week prior to the start of the experiments. All procedures followed were in accordance with the standards set forth in the *Guide for the Care and Use of Laboratory Animals* and were approved by the animal ethics committee of Shandong University. C57BLKS/J db/m mice were selected as control group (CC, n = 8). The db/db mice were divided into 2 groups: an untreated diabetic group (DM, n = 8) and GSPB2-treated group (30 mg/kg body weight/day, DMT, n = 8). GSPB2 was given diluted in normal saline solution by intragastric administration for 10 weeks. Each group of mice was observed from week 7 to week 18 without any hypoglycemic therapy. At the end of the experiments, all mice were fasted overnight and then sacrificed under sodium pentobarbital anesthesia. Fasting blood was collected, and the aortas were dissected. Aortic tissues and sera were kept at −80°C until further analysis.

### Estimation of Body Weight, Fasting Blood Glucose (FBG), Blood Lipid and Advanced Glycation End Products (AGEs)

Animals were weighed every week. FBG, total cholesterol (TC), triglyceride (TG) levels were determined by DVI-1650 Automatic Biochemistry and Analysis Instrument (Bayer, Germany). Serum AGEs specific fluorescence determinations were performed by measuring emission at 440 nm on excitation at 370 nm using a fluorescence spectrophotometer (HITACHI F-2500, Japan) at the end of the treatment.

### Proteomic Analysis

#### Mass spectrometric analysis of iTRAQ samples

Mass spectrometric analysis was performed using a micro liquid chromatography system (MDLC, GE Healthcare) and a LTQ-Velos ion trap mass spectrometer (Thermo Finnigan, San Jose, CA, USA). The separation column was a 0.15 mm×150 mm capillary packed with Zorbax 300SB-C18particles (Agilent Technologies). Mobile phase A (0.1% formic acid in water) and the mobile phase B (0.1% formic acid in ACN) were selected. The volumetric flow rate in the separation column was set to about 1 µl/min, with a 2 h long separation gradient running from 0% to 100% B.

Mass spectrometric data were acquired using data-dependent acquisition conditions: each MS event was followed by zoom/MS^2^ scans on the five top-most intense peaks; zoom scan width was ±5 m/z; dynamic exclusion was enabled at repeat count 1, repeat duration 30 s, exclusion list size 200, exclusion duration 60 s, and exclusion mass width ±1.5 m/z; PQD parameters were set at isolation width 2 m/z, normalized collision energy 35%, activation Q 0.7, and activation time 0.1 ms; the threshold for MS/MS acquisition was set to 500 counts.

#### Data analysis

For protein identification and statistical validation, the acquired MS/MS spectra were automatically searched against the non-redundant International Protein Index (IPI) mouse protein database (version 3.72) using the Turbo SEQUEST program in the BioWorksTM 3.1 software suite. The database search parameters included the following settings: number of allowed missed tryptic cleavage sites was set to 2, the peptide tolerance was 2 u, the fragment ion tolerance was 1 u, and only fully tryptic fragments were considered for peptide selection. The sensitivity threshold and mass tolerance for extracting the iTRAQ ratios were set to 1 and ±0.5, respectively. Data filtering parameters were chosen to generate false positive protein identification rates of <1%, as calculated by searching the MS^2^ scans against a forward reversed database of proteins. A threshold was set to 1.5 with a *P*-value <0.05 yielding at least a 50% change in abundance compared to the reference (CC group).

#### Subcellular localization analysis and functional classification

The localization analysis of the identified proteins in aortas was performed by using AmiGO (Version 1.8). We get the details including the information of subcellular localization by manual input the name of these proteins.

Sequences of all identified proteins by iTRAQ were submitted to KOGnitor for KOG (eukaryotic orthologous groups) classification (http://www.ncbi.nlm.nih.gov/COG/grace/kognitor.html) [16]. When we manual input an identified protein sequence, it can be assigned with a KOG number. A KOG number belongs to one category. The protein ratio for each category was calculated by dividing the number of proteins within a category by the sum of assigned proteins from all categories.

#### Protein network analysis

The global protein changes data in the aorta of db/db mice treatment with GSPB2 were analyzed through the use of Ingenuity Pathways Analysis (Ingenuity Systems, CA). This program uses a knowledge database derived from literature to relate gene products based on their interaction and function. The proteins with their abundance change and corresponding Swiss-Prot accession numbers were imported into the program. Ingenuity software uses the data to navigate the Ingenuity pathways database for interactions between these focus proteins and all the other protein stored in the database to generate biological networks. A score better than 2 is usually considered as a valid network.

### ELISA of Serum MFG-E8 in the Patients with T2DM

#### Human studies

Fifty five elderly patients with T2DM (mean age 69.36±7.44 years) were registered as outpatients in the Department of Geriatrics of Qi-Lu Hospital of Shandong University, China. The inclusion criteria were the presence of T2DM according to the 1999 diagnostic guidelines of the World Health Organization (WHO), given routine pharmacotherapy with an oral hypoglycemic agent and/or insulin for at least one year. We excluded patients who had a history of alcohol abuse, evidence of liver disease, or severe cardiac problems and dialysis. All patients signed informed consent. The study protocol was approved by Qi-Lu Hospital of Shandong University Ethics Board and was conducted according to the Declaration of Helsinki (1964). CF-PWV was measured by an automatic device (Complior, France).

#### ELISA of MFG-E8 in human serum

A 96-well microplate (NUNC plate) was coated with 0.2 µg/well of monoclonal anti-human lactadherin antibody (R&D Systems) in PBS buffer (pH 7.4, 137 mM NaCl, 2.7 mM KCl, 8.1 mM Na_2_HPO4, 1.5 mM KH2PO4). A 100 µL of human serum sample in diluent (1% BSA in PBS, pH7.4) buffer was added to each well. After an incubation period of 2 h at room temperature, the wells were aspirated and washed with wash buffer (0.05% Tween 20 in PBS, pH 7.4). Each well was then incubated with 2 µg/mL lactadherin (rabbit polyclonal IgG from Santa Cruz) in diluent buffer for 2 h at room temperature, washed as above and incubated with 0.4 µg/mL of goat anti rabbit IgG-HRP (Santa Cruz) for 20 min at room temperature. HRP activity was determined using a microplate reader set to 450 nm with 570 nm wavelength correction. Recombinant human lactadherin (R&D systems) was used to prepare the standard curve.

### Treatment of MFG-E8 RNAi and Recombinant MFG-E8 in db/db Mice

#### Primary culture of aortic endothelial cell

The aorta of male C57BLKS/J db/m mice was isolated. Longitudinal dividing the aortic wall and cut into small pieces, stick the lining surface in the cell culture flasks. Then put it in the incubator with endothelial cell medium (ECM, ScienCell) with an atmosphere of 5% CO_2_/95% air. After 7–9 days, cell fused into a single layer, dealt with 0.25% trypsin digestion and passage. Cells from passages 3 to 6 were used in this study.

#### Construction of MFG-E8 RNAi and endothelial cell transfection

Lentiviral vector with short hairpin RNAs (shRNAs) for MFG-E8 were designed and chemically synthesized from Shanghai GeneChem Co., Ltd (Shanghai, China). The lentiviral vector with shRNA sequence targeting MFG-E8 (GeneBank accession no: NM_001045489) including: sense: 5′-CcggGCTGGATAATCAGGGCAAGATTTCAAGAGAATCTTGCCCTGATTATCCAGCTTTTTg-3′; antisense: 5′-aattcaaaaaGCTGGATAATCAGGGCAAGATTCTCTTGAAATCTTGCCCTGATTATCCAGC-3′. The lentiviral vector with green fluorescence protein (GFP) was used as the RNAi control.

Aortic endothelial cell were transfected with lentiviral vectors at a multiplicity of infection (MOI = 100) according to the manufacturer’s instructions. Fluorescence microscope was used to measure transfection efficiency at time points of 12, 24, 48 and 72 hours after transfection. The most effective target site of RNA interference was confirmed by using western blotting for the expression levels of MFG-E8 after 3 days.

#### Animals and groups

Male C57BLKS/J db/db and db/m mice (n = 40, 7 weeks old) were used in this study. C57BLKS/J db/m mice were selected as control group (CC, n = 8). The db/db mice were divided into 4 groups: an untreated diabetic group (DM, n = 8), LV-GFP treated db/db group (GFP, n = 8), MFG-E8 RNAi treated db/db mice group (M-RNAi, n = 8) and recombinant MFG-E8 treated db/db mice group (rmMFG-E8, n = 8). Each group of mice was observed from week 13 to week 18 without any administration of hypoglycemic therapy. At the end of the intervention, all mice were fasted overnight and then sacrificed. Fasting blood was collected, and the aortas were dissected. Aortic tissues and sera were kept at −80°C until further analysis.

#### Treatment of MFG-E8 RNAi and recombinant MFG-E8 in db/db mice

After testing knockdown efficiencies of the four shRNA sequences by using aortic endothelial cells, the most efficient sequences targeting MFG-E8 or LV-GFP was diluted to a total volume of 300 ul containing 4×10^7^ TU was injected into the tail vein of fourteen-week-old male db/db mice. Recombinant mouse MFG-E8 (R&D Systems) was diluted in PBS and 300 ul of the solution (20 ug/kg) was injected through the tail vein of fourteen-week-old male db/db mice twice a week for 4 weeks. After 4 weeks of treatment, each mouse of M-RNAi group, GFP group and rmMFG-E8 group was perfused with ice-cold physiological saline (0.9% NaCl, pH 7.4) after cardiac puncture, and the aortas were excised, placed in liquid nitrogen, and stored at −80°C until analysis.

### Morphology Examination

Aortas were fixed in 4% paraformaldehyde and embedded in paraffin, and 5 µm thick sections were cut. Then stained with hematoxylin-eosin and examined by light microscopy. The intima-media thickness was measured using Image Proplus 6.0.

Part of aorta was fixed in 3% glutaraldehyde in cacodylate buffer at 4°C for 2 h and subsequently post fixated in a 1% osmium-tetroxide phosphate buffer solution for 1 h. Next, the samples were dehydrated in a graded ethanol series with acetone, permeated, and embedded in epoxide resin. Ultrathin sections were stained with uranylacetate and lead citrate, and examined under an H-800 electron microscope (TEM, Hitachi Electronic Instruments, Tokyo, Japan).

### Western Blotting Analysis

Western blotting analysis was performed on samples of aortas obtained from six groups of mice. Equal amount of proteins were separated by electrophoresis in a SDS-polyacrylamide gel. After the proteins were transferred onto a polyvinylidene difluoride membrane, the blot was incubated with blocking buffer (5% non-fat dry milk and 0.05% Tween 20 in TBS) for 1 h at room temperature and then probed with antibody for MFG-E8 (1∶1000 dilution; R&D), phosphorylated ERK1/2 (1∶1000 dilution; Cell Signaling), CSRP1 (1∶1000 dilution; Abcam) and GSTT1 (1∶1000 dilution; Santa Cruz) overnight at 4°C. After incubation in horseradish peroxidase-conjugated secondary antibody, the immunoblots were visualized with ECL immunoblotting detection system, then stripped and reprobed with antibody recognizing β-actin (1∶1000 dilution; Cell Signaling), ERK1/2 (1∶1000 dilution; Cell Signaling) and GAPDH (1∶1000 dilution; Cell Signaling) to ensure equal loading. The intensity of each protein band was quantified with densitometric analysis.

### ELISA of MCP-1 in db/db and db/m Mice Serum

A 96-well microplate was coated with monoclonal antibody to mouse MCP-1 (eBioscience). A 100 µL of mouse serum sample in diluent buffer was added to each well. After an incubation period of 2 h at room temperature, the wells were aspirated and washed with wash buffer. A biotin-conjugated anti-mouse MCP-1 antibody was added and binds to mouse MCP-1 captured by the first antibody, then washed as above and incubated with streptavidin-HRP for 1 h at room temperature. Following incubation unbound Streptavidin-HRP was removed during a wash step, and substrate solution reactive with HRP was added to the well. A colored product was formed in proportion to the amount of mouse MCP-1 present in the mouse serum sample. The reaction was terminated by addition of acid and absorbance was measured at 450 nm. A standard curve was prepared from 7 mouse MCP-1 standard dilutions and mouse MCP-1 sample concentration determined.

### Statistical Analysis

Data were expressed as mean ± standard deviation. Statistical analysis between groups was made using one-way analysis of variance (ANOVA) followed by Tukey's HSD test for multiple comparisons. *P*-value <0.05 was considered statistically significant. All analyses were performed with SPSS for Windows software version 10.0 (SPSS, Chicago, USA).

## Results

### Effects of GSPB2 on Body Weight, FBG, AGEs, TG and TC

**Figure 1:**
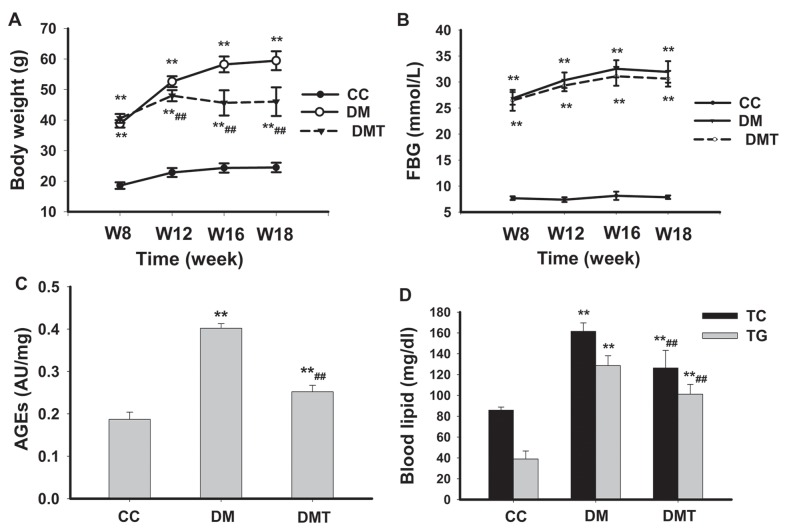
Effects of GSPB2 on body weight, FBG, AGEs, TG and TC in db/db mice. **A:** Body weight changes of the mice. **B:** FBG changes of the mice. **C:** Measurement of AGEs 10 weeks after initiation of the experiment. **D:** Measurement of serum TG and TC 10 weeks after initiation of the experiment. **P<0.01 compared with CC group; ^##^ *P*<0.01 compared with DM group. CC: control db/m group; DM: untreated db/db group; DMT: GSPB2 treated db/db group. GSPB2: grape seed procyanidin B2; FBG: fasting blood glucose; AGEs: advanced glycation end products; TG: triglyceride; TC: total cholesterol.

Mice were maintained over the length of this study without any hypoglycemic treatments. Figure **1A** shows body weight among the control, untreated and GSPB2-treated diabetic mice. The body weight of diabetic mice was significantly higher than that of the control mice at 8 weeks, 12 weeks, 16 weeks, and 18 weeks (*P*<0.01). GSPB2 significantly decreased the body weight of diabetic mice at 12 weeks, 16 weeks, and 18 weeks (*P*<0.01). Figure **1B** shows FBG among the control, untreated and GSPB2-treated diabetic mice. The FBG of diabetic mice was significantly higher than the control mice at 8 weeks, 12 weeks, 16 weeks, and 18 weeks (*P*<0.01), but GSPB2 did not decrease FBG at 8, 12, 16, and 18 weeks (*P*>0.05). The serum AGEs, TG and TC of diabetic mice at 18 weeks were higher than those of the control mice (*P*<0.01). GSPB2 significantly reduced the serum AGEs, TG and TC of diabetic mice (*P*<0.01) (Figure **1C, 1D**).

### Quantification and Identification of the Aortic Proteome by iTRAQ Analysis

To further investigate the effects of GSPB2 on db/db mice, we used iTRAQ to generate the protein expression profiles of four aorta samples per group. In total, 557 proteins were shown to have significantly different abundance between control group and DM group (±1.5-fold). Of these 557 proteins, the levels of 139 proteins were normalized by GSPB2 treatment. Table **S1** showed the proteins identified, including the name, accession number, function, molecular weight, PI, and protein expression ratio. In brief, the differentially expressed proteins that were normalized by GSPB2 were related to many important biological functions including oxidative stress, metabolism, apoptosis, tissue remodeling, and heat shock. MFG-E8, the protein which we further investigated, was increased by 2.4-fold in abundance in db/db mice, which was normalized by GSPB2 treatment (protein expression ratio: DMT/DM 0.59; DMT/CC 1.43). Eight unique peptides from MFG-E8 were identified, and the cover percent of protein was 22.46%.

### Subcellular Localization Analysis, Bioinformatic Functional Analysis and Ingenuity Pathway Analysis of Differentially Abundant GSPB2 Associated Arterial Proteins in db/db Mice

**Figure 2:**
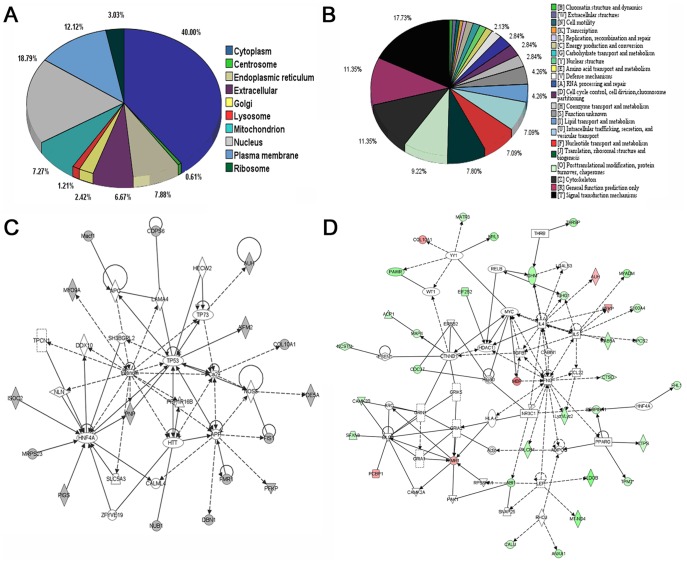
Subcellular localization, functional classification analysis and ingenuity pathway analysis of the identified differential proteins between untreated db/db mice and GSPB2 treated db/db mice. A: Subcellular localization by AmiGO analysis of the identified differential proteins between untreated db/db mice and GSPB2 treated db/db mice. **B:** Functional classification by KOG analysis of the identified differential proteins between untreated db/db mice and GSPB2 treated db/db mice. **C** and **D:** Ingenuity pathway analysis (score 46) based on the differentially expressed aortic proteins between untreated db/db mice and GSPB2 treated db/db mice. Protein nods with colored background correspond to the identified proteins. GSPB2: grape seed procyanidin B2.

The localization analysis of the identified proteins in aorta using AmiGO (Version 1.8) is shown in Figure **2A**. Among these proteins, some are located in one or more subcompartments of the cell. 40% were in cytoplasm, 18.8% in nucleus, 12.1% in plasma membrane, 7.9% in endoplasmic reticulum, 7.3% in mitochondrion, 6.7% in extracellular, 6% in centrosome, 3% in ribosome, 2.4% in Golgi, 1.2% in lysosome.

The functional classification of the identified proteins in aortas is shown in Figure **2B**. Among the functional assignment of the proteins, 17% were involved in signal transduction mechanisms, 12% in cytoskeleton, 9% in posttranslational modification, protein turnover, chaperones, 7% in translation, ribosomal structure and biogenesis, 6% in intracellular trafficking, secretion, and vesicular transport, 4% in cell cycle control, cell division, chromosome partitioning, 4% in nucleotide transport and metabolism, 4% in lipid transport and metabolism, and the remaining proteins were identified as dispersed across 13 remaining categories.

Ingenuity Pathway Analysis (IPA) (http://www.ingenuity.com) shows that the primary pathway involved was cell death, lipid metabolism and small molecule biochemistry, where 16 of the proteins could be linked together (Figure **2C**). The proteins include Isoform 1 of Apoptosis-inducing factor 2 (AIFM2), Mitochondrial fission 1 protein isoform 2 (FIS1), Isoform 1 of microtubule-actin cross-linking factor 1 (Macf1), Isoform 1 of methylglutaconyl-CoA hydratase (AUH), Platelet isoform of phosphofructokinase (PFKP), and so on. The expression of these proteins was decreased in diabetic aorta, and was normalized by GSPB2. Together with Interleukin 4 (IL-4), Interleukin 5 (IL-5), Interferon-γ (IFNγ), Peroxisome proliferator-activated receptor-γ (PPARγ), Leptin (LEP), Heat shock protein-90 (Hsp90), Transforming growth factor-β (TGFβ), and other molecules, the differentially expressed proteins normalized by GSPB2 form a network of cell death and lipid metabolism (Figure **2D**). The pathways would facilitate the understanding of diabetes biomarkers for further study.

### Serum MFG-E8 in the Patients with T2DM

**Figure 3:**
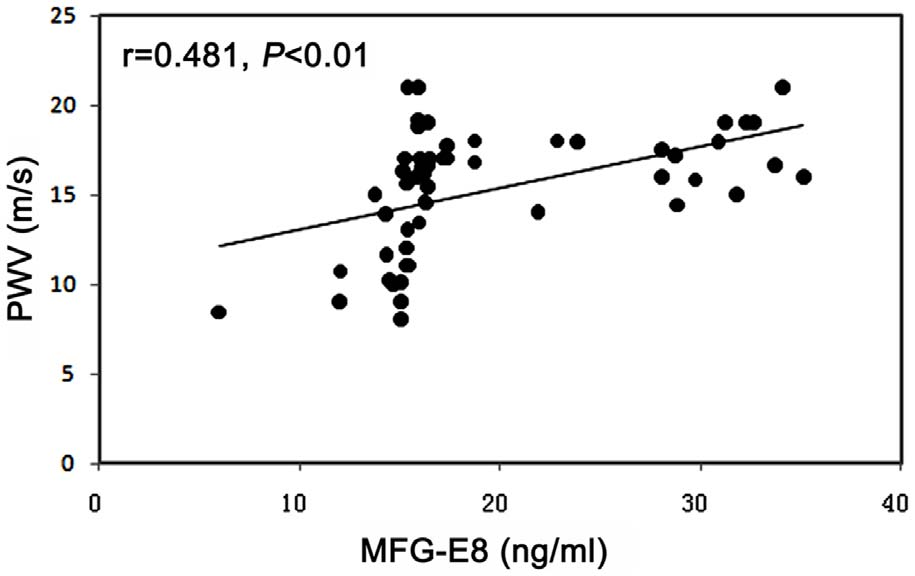
Correlation between serum MFG-E8 and CF-PWV in T2DM patients (n = 55). MFG-E8: milk fat globule epidermal growth factor-8; CF-PWV: carotid- femoral pulse wave velocity.

We examined the serum MFG-E8 of 55 T2DM patients by using ELISA to determine the clinical significance of the proteomic findings of the raised MFG-E8 in the aorta of db/db mice. We observed the increased serum MFG-E8 significantly correlated with increased aortic stiffness measured by PWV (r  = 0.481, *P*<0.01) (Figure **3**).

### Transfection Efficiency with MFG-E8 RNAi

**Figure 4:**
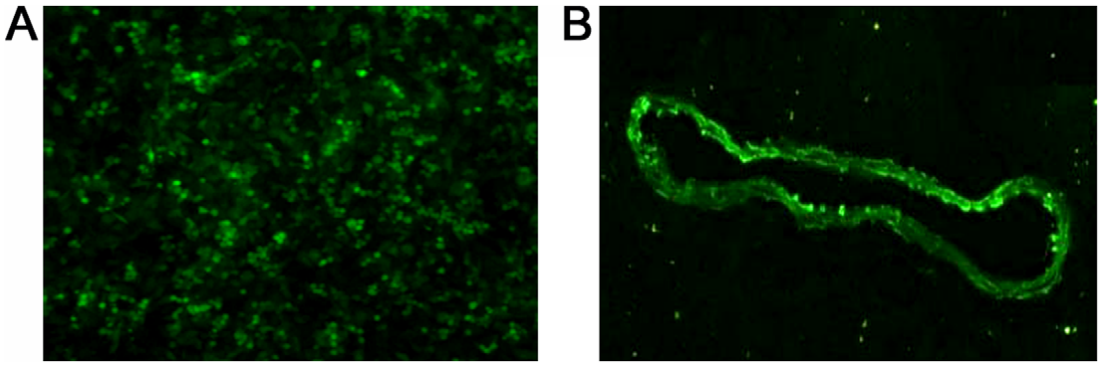
GFP transfection in aortic endothelial cells and aorta of db/db mice. **A:** Fluorescence micrograph displays GFP in aortic endothelial cells (×200). **B:** Fluorescence micrograph displays GFP in aorta of db/db mice (×200).

The aortic endothelial cells were treated with MFG-E8 RNAi or lentivirus vector. Transfection conditions were optimized by using different MOI and the transfection efficiency was assessed by fluorescence microscope and western blotting. Aortic endothelial cell carrying GFP was observed with fluorescence microscope (Figure **4A**). The transfection efficiency was about 95% at 72 hour or longer (MOI  = 100).

To investigate the transfection efficiency of RNAi *in vivo*, we transfected db/db mice with lentiviral vector carrying GFP injected into the tail vein. After 7 days, the aorta were excised and observed by fluorescence microscope (Figure **4B**).

### Histological Findings

**Figure 5:**
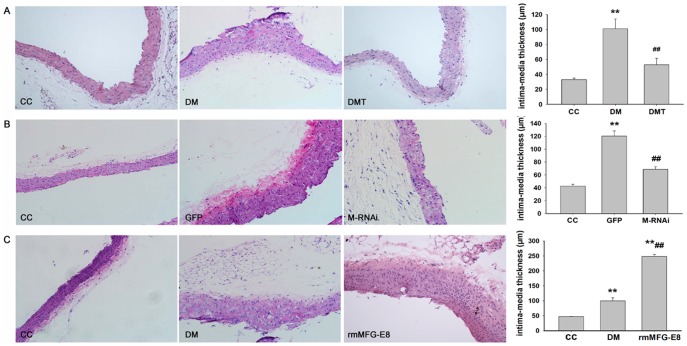
Representative light micrographs of the aorta (hematoxylin-eosin; ×200). **A:** Effects of GSPB2 on the aorta of db/db mice. **B:** Effects of MFG-E8 RNAi on the aorta of db/db mice. **C:** Effects of recombinant MFG-E8 on the aorta of db/db mice. Statistical results of intima-media thickness were shown in bar graph. **P<0.01 compared with CC group; ^##^ *P*<0.01 compared with DM or GFP group. CC**:** control db/m group; DM**:** untreated db/db group; DMT: GSPB2 treated db/db group**;** GFP: treatment of GFP in db/db group; M-RNAi: treatment of MFG-E8 RNAi in db/db group; rmMFG-E8: treatment of recombinant MFG-E8 in db/db group.

Under light microscopy, aortic remodeling and proliferation of vascular smooth cells (VSMC) and endothelial injury were observed in the aorta of db/db mice. Moreover, GSPB2 and MFG-E8 RNAi suppressed the endothelial injury, aortic remodeling and proliferation of VSMC and led to light microscopic findings similar to those of the control mice (Figure **5A** and **5B**). In aorta of recombinant MFG-E8 protein-treated group, the deteriorative changes were observed. The blood vessel was thicker, characterized by more inflammatory cells infiltrated such as neutrophil and lymphocyte and focal necrosis. Endothelial cell swelling and perivascular inflammation were also observed (Figure **5C**).

**Figure 6:**
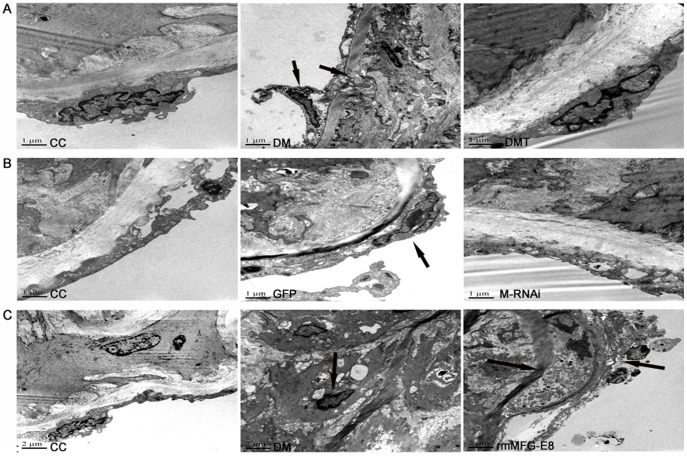
Representative Electron Micrographs of the aorta (scale bar: 1 µm or 2 µm). **A:** Effects of GSPB2 on the aorta of db/db mice. **B:** Effects of MFG-E8 shRNA on the aorta of db/db mice. **C:** Effects of recombinant MFG-E8 on the aorta of db/db mice. Black arrows show abnormal ultrastructure changes. CC**:** control db/m group; DM**:** untreated db/db group; DMT: GSPB2 treated db/db group**;** GFP: treatment of GFP in db/db group; M-RNAi: treatment of MFG-E8 shRNA in db/db group; rmMFG-E8: treatment of recombinant MFG-E8 in db/db group.

Under electron microscopy, the impaired endothelial cells and the rod-like mitochondria in endothelial cells were seen in aortic tissue in the diabetic or GFP treated mice. Moreover, the rupture of vascular elastic membrane and the increased collagen fibers were observed in the diabetic aorta. Many smooth muscle cells showed pyknotic nucleus, increased heterochromatin, mitochondrial condensation, swelling of the endoplasmic reticulum, and many autophagosome appeared, whereas normal ultrastructure was observed in the aortic tissue of the control db/m mice. GSPB2 and MFG-E8 RNAi tended to improve the preservation of the fine structure of aortic tissue (Figure **6A** and **6B**), but in the rmMFG-E8 group, the deteriorative changes and the migration of smooth muscle cells in the vascular elastic membrane were observed (Figure **6C**).

### Effects of GSPB2, MFG-E8 RNAi and Recombinant MFG-E8 on the Expression of MFG-E8 in db/db Mice

**Figure 7:**
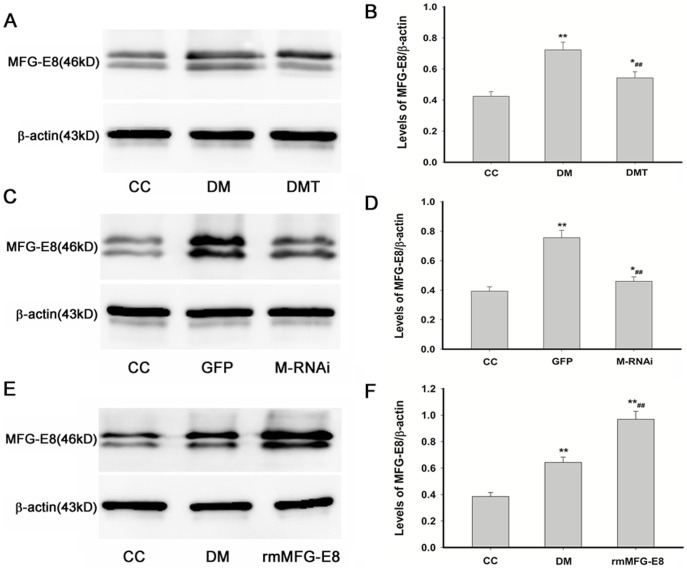
Effects of GSPB2, MFG-E8 RNAi and recombinant MFG-E8 on the expression of MFG-E8 in db/db mice by Western blotting analysis. Data were expressed as the expression ratio of MFG-E8/β-actin and given as mean ± SD from three experiments. **P<0.01, *P<0.05 compared with CC group; ^##^ *P*<0.01 compared with DM or GFP group. CC: control db/m group; DM: untreated db/db group; DMT: GSPB2 treated db/db group; GFP: treatment of GFP in db/db group; M-RNAi: treatment of MFG-E8 shRNA in db/db group; rmMFG-E8: treatment of recombinant MFG-E8 in db/db group.

In order to confirm the results of iTRAQ, the protein expression of MFG-E8 in the aorta of control db/m mice, untreated db/db mice, and GSPB2 treated db/db mice was carried out by Western blotting analysis. The protein expression of MFG-E8 in db/db mice were significantly higher than that of control db/m mice (*P*<0.01); after treatment with GSPB2, the expression of MFG-E8 was decreased compared to that of untreated db/db mice (*P*<0.01), which was in accordance with the result of iTRAQ (Figure **7A** and **7B**).

Western blotting analysis showed protein expression of MFG-E8 in GFP group was significantly higher than that of control db/m group (*P*<0.01); after treatment with MFG-E8 RNAi, the expression of MFG-E8 decreased compared to GFP treated db/db mice (Figure **7C** and **7D**), which confirmed the transfection efficiency in db/db mice in vivo.

Figure **7E** and **7F** show the recombinant MFG-E8 on the protein expression in db/db mice. The protein expression of MFG-E8 in db/db mice was significantly higher than that of control db/m mice (*P*<0.01); however, the rmMFG-E8 group displayed increased expression of MFG-E8 compared to that of both control db/m group and DM group (*P*<0.01).

### Effects of GSPB2, MFG-E8 RNAi and Recombinant MFG-E8 on ERK1/2 Phosphorylation Levels in db/db Mice

**Figure 8:**
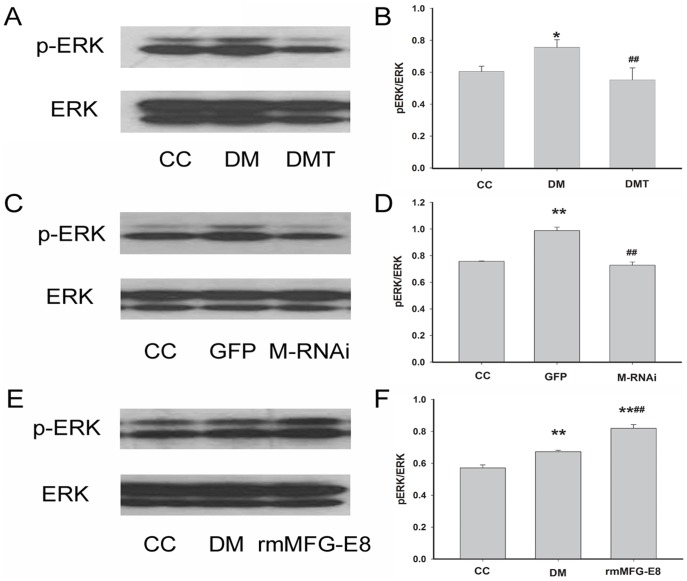
Effects of GSPB2, MFG-E8 RNAi and recombinant MFG-E8 on the expression of p-ERK in db/db mice by Western blotting analysis. Data were expressed as the expression ratio of p-ERK/total-ERK and given as mean ± SD from three experiments. **P<0.01, *P<0.05 compared with CC group; ^##^ *P*<0.01 compared with DM or GFP group. CC: control db/m group; DM: untreated db/db group; DMT: GSPB2 treated db/db group; GFP: treatment of GFP in db/db group; M-RNAi: treatment of MFG-E8 shRNA in db/db group; rmMFG-E8: treatment of recombinant MFG-E8 in db/db group.

To understand the mechanism of atherosclerosis in T2DM, we further examined the ERK1/2 phosphorylation (p-ERK1/2) in aorta of db/db mice by Western blotting. The expression of p-ERK1/2 in db/db mice were higher than that of control db/m mice (*P*<0.05); after treatment with GSPB2, the expression of p-ERK1/2 was decreased compared to that of untreated db/db mice (Figure **8A** and **8B**). MFG-E8 RNAi decreased in the phosphorylated ERK1/2 (*P*<0.01) (Figure **8C** and **8D**). Treatment with recombinant MFG-E8 increased ERK1/2 phosphorylation (*P*<0.01) (Figure **8E** and **8F**).

### Effect of GSPB2, MFG-E8 RNAi and Recombinant MFG-E8 on the Levels of MCP-1 in db/db Mice

**Figure 9:**
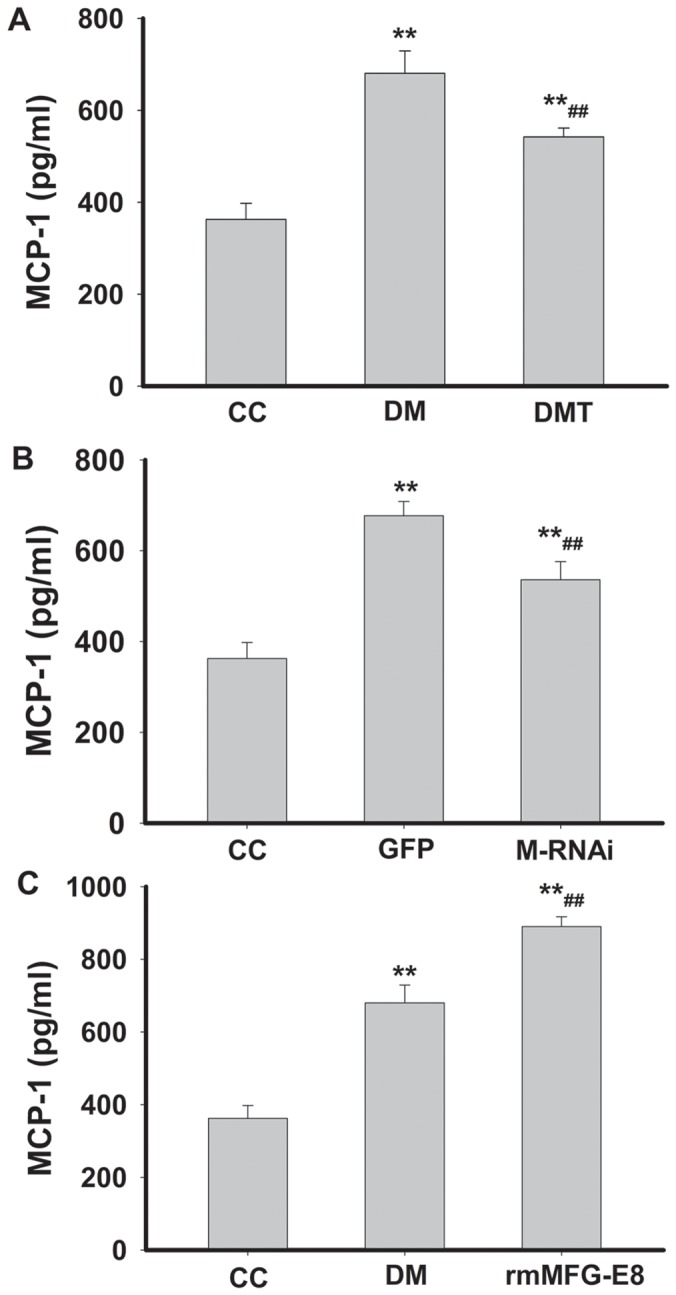
Effects of GSPB2, MFG-E8 RNAi and recombinant MFG-E8 on the expression of MCP-1 in serum of db/db mice by ELISA. **P<0.01 compared with CC group; ^##^ *P*<0.01 compared with DM or GFP group. CC**:** control db/m group; DM**:** untreated db/db group; DMT: GSPB2 treated db/db group**;** GFP: treatment of GFP in db/db group; M-RNAi: treatment of MFG-E8 shRNA in db/db group; rmMFG-E8: treatment of recombinant MFG-E8 in db/db group.

Figure **9** shows the effects of GSPB2, MFG-E8 RNAi and recombinant MFG-E8 on the expression of MCP-1 in serum of db/db mice by ELISA. The expression of MCP-1 in serum was significantly increased in DM group compared with that of control group (*P*<0.01). GSPB2 and MFG-E8 RNAi decreased the level of MCP-1 (*P*<0.01) (Figure **9A** and **9B**). Recombinant MFG-E8 significantly increased the expression of MCP-1 (*P*<0.01) (Figure **9C**).

### Validation of iTRAQ Data on Other Selected Proteins

To validate the proteomic analysis of aorta using iTRAQ we performed, CSRP1 and GSTT1 were validated using western blotting analysis. CSRP1 was found to be inhibited whereas GSTT1 was enhanced in the DMT group compared to the DM group (Figure **S1**). The significance of those proteins would be interesting topic for our future studies.

## Discussion

Atherosclerotic diseases are among the major complications of T2DM leading to morbidity and mortality. The mechanism underlying atherogenesis in T2DM has been under extensive investigation. Studies have shown that oxidative stress plays a key role in this process [17]–[20]. Reactive oxygen species (ROS) can lead to damage of endothelial cells and to proliferation, migration and phenotypic change of smooth muscle cells, contributing to atherogenesis in the setting of DM [21], [22].

GSPE has potent antioxidant effects [23]–[28]. Studies have shown that GSPE have anti-atherosclerotic effects both in animals and human subjects, which has been closely correlated with its antioxidant potency [29]. Our previous studies showed that GSPE demonstrated anti-non-enzymatic glycation and anti-inflammatory effects in DM animal models [30], [31]. GSPB2 is one of the main components of GSPE. Our studies showed that GSPB2 has protective effects against early stage endothelial dysfunction in DM.We also reported for the first time, to the best of our knowledge, that GSPB2 inhibited the AGE-induced proliferation and migration of human aortic smooth muscle cells (HASMCs) through inhibiting the nuclear translocation of nuclear factor-κB and the degradation of its inhibitor, IκB-α [15]. Notwithstanding the extensive molecular studies the data regarding the proteomic profile of aorta in DM and the effect of pharmacological intervention with respect to the proteomic changes has been scarce.

In this study, our results showed that GSPB2 significantly prevented the development of obesity in db/db mice during the 18-week experimental period. GSPB2 also prevented the elevation in the levels of serum AGEs observed in db/db mice, suggesting an anti-non-enzymatic glycation effect of GSPB2. Our *in vivo* data showed that GSPB2 inhibited hyperlipidemia in db/db mice. VSMC proliferation and endothelial cell damage were suppressed by GSPB2 in the aortic wall of db/db mice (Figure **5** and **6**). Although the glucose level was not significantly decreased by the administration of GSPB2 [32], a tendency to reduce the glucose level was observed.

iTRAQ was used to investigate aorta protein profiles among db/m, untreated db/db, and GSPB2 treated db/db mice. More than 1500 proteins were detected, of which 557 demonstrated significant changes in the expression levels in DM aorta, and 139 proteins of them were normalized by GSPB2 administration.

Some proteins contributing to cytoskeletal of the vasculature were found up-regulated in the aorta of db/db mice and down-regulated in the aorta of GSPB2-treated db/db mice. CSRP1 is expressed primarily in VSMCs and in sensing or responding to pathological vascular stress and maintaining smooth muscle homeostasis [33]. It is thought to be critical for smooth muscle differentiation. Our results suggested that the inhibition of CSRP1 partly contributed to the improvement of aortic damage.

Moreover, it is well known that increased oxidative stress has been implicated in the pathogenesis of diabetic vascular complications of type 2 diabetes [34]. Our data showed that glutathione S-transferase, theta 1 (GSTT1), an enzyme actively involved in alleviating oxidative stress was down-regulated in the db/db mice aorta which was reversed by GSPB2 treatment. GSTT1 is associated with the regulation of inflammation through modulation of prostaglandin signaling pathways and oxidative stress [35]. Decreased GSTT1 expression contributes to increased oxidative stress in the db/db mice.

MFG-E8, also known as lactadherin, plays an important role in the pathogenesis of diabetic vascular-related process. In aorta, MFG-E8 is a protein mainly expressed by adventitial microvessels, medial smooth muscle cells and luminal endothelial cells [36]. Our previous proteomic studies showed that the expression of MFG-E8 in the aorta of type 1 diabetic rats was significantly higher than in control rats and treatment with GSPE significantly inhibited the expression of MFG-E8 in diabetic rats [37], suggesting that MFG-E8 was involved in oxidative stress and inflammatory process in diabetic aortic disease. MFG-E8 was up-regulated with increased oxidative. It has recently been reported that high concentration of glucose up-regulated MFG-E8 in the adiposomes, increased ROS production [38]; ROS scavenger N-acetyl cysteine decreased MFG-E8 in the adiposomes. Expression of MFG-E8 was up-regulated in epididymal adipose tissues of diet-induced obese C57BL/6 mice as well as in those of the genetically obese ob/ob and db/db mice [39]–[41]. In this study we found that MFG-E8 protein was increased by 2.4-fold in abundance in db/db mice as compared with db/m mice, which was reversed by GSPB2 treatment. Given the increased aortic expression of MFG-E8 in both type 1 and 2 diabetic animal models, we further investigated the significance of MFG-E8 in the development of atherosclerosis in DM as a target of GSPB2 among the identified differential proteins.

Inhibition of MFG-E8 expression by RNAi significantly reduced whereas exogenous administration of recombinant MFG-E8 promoted the pathological processes involved in atherogenesis in db/db mice, including endothelial cell injury, VSMC proliferation and aortic remodeling (see Figure **5** and **6**). Interestingly we also found that serum MFG-E8 level is positively correlated with increased PWV in T2DM patients, the increase of which is a well-established parameter for increased arterial stiffness. This finding is consistent with our previous data that serum MFG-E8 level was elevated in diabetic patients as compared with that in healthy subjects [42].

ERK1/2 activation has been reported to play a pivotal role in cell replication in the arterial wall after injury [43], and associated with the proliferation of HASMCs [44]. ERK1/2 activation/phosphorylation is also involved in the increased production of reactive oxygen species and the toxic effects of AGEs, processes involved in atherogenesis in DM [45], [46]. The present study showed that ERK1/2 phosphorylation expression was suppressed in aorta of GSPB2-treated mice. Targeted inhibition of MFG-E8 by RNAi suppressed while exogenous recombinant MFG-E8 led to increased ERK1/2 phosphorylation. We also found that GSPB2 treatment reduced serum level of MCP-1, a potent chemokine in the pathogenesis of inflammation and atherosclerosis [47], [48], in the db/db mice as a result of inhibition of MFG-E8. Circulating MCP-1 has been implicated as causative factors in the development of T2DM. MCP-1 activates ERK1/2 in endothelial cells and pancreatic β cells [49]–[51]. These findings demonstrate that MFG-E8 induces atherosclerosis in db/db mice through ERK1/2 and MCP-1 signaling pathways. Therefore, further studies are needed to clarify the cross-talk and mechanism between these biomarkers. Although GSPB2 is a well-studied antioxidant, whether and how the inhibitory effect of GSPB2 on atherosclerosis in db/db mice is related to its antioxidative effects will be the topic of our studies to come.

### Conclusion

In conclusion, our study shows that MFG-E8 plays an important role in the early arterial damages in atherogenesis in diabetes. GSPB2 inhibits atherogenesis in db/db mice, at least in part, due to its inhibition of aortic expression and serum level of MFG-E8. Measuring MFG-E8 serum level could be a useful clinical surrogate prognosticating atherogenesis in DM patients. Approaches targeting MFG-E8 including use of GSPB2 could potentially lead to new modality in the prevention and treatment of vascular complications in DM patients.

## Supporting Information

Figure S1 Western blotting validation of iTRAQ data on other two proteins: CSRP1 and GSTT1. GAPDH was used as the loading control. CC**:** control db/m group; DM**:** untreated db/db group; DMT: GSPB2 treated db/db group. **P<0.01 compared with CC group; ^##^ *P*<0.01 compared with DM group.
(TIF)

Materials and Methods S1 (DOCX)

Table S1 Differentially expressed proteins as reversed by GSPB2 identified by iTRAQ
(DOC)
