# Supplementary figures and images for: Proteomic Analysis of Aorta and Protective Effects of Grape Seed Procyanidin B2 in db/db Mice Reveal a Critical Role of Milk Fat Globule Epidermal Growth Factor-8 in Diabetic Arterial Damage
Source: PLoS One. 2012 Dec 21;7(12):e52541. doi: 10.1371/journal.pone.0052541 (PMC3528673; doi:10.1371/journal.pone.0052541)

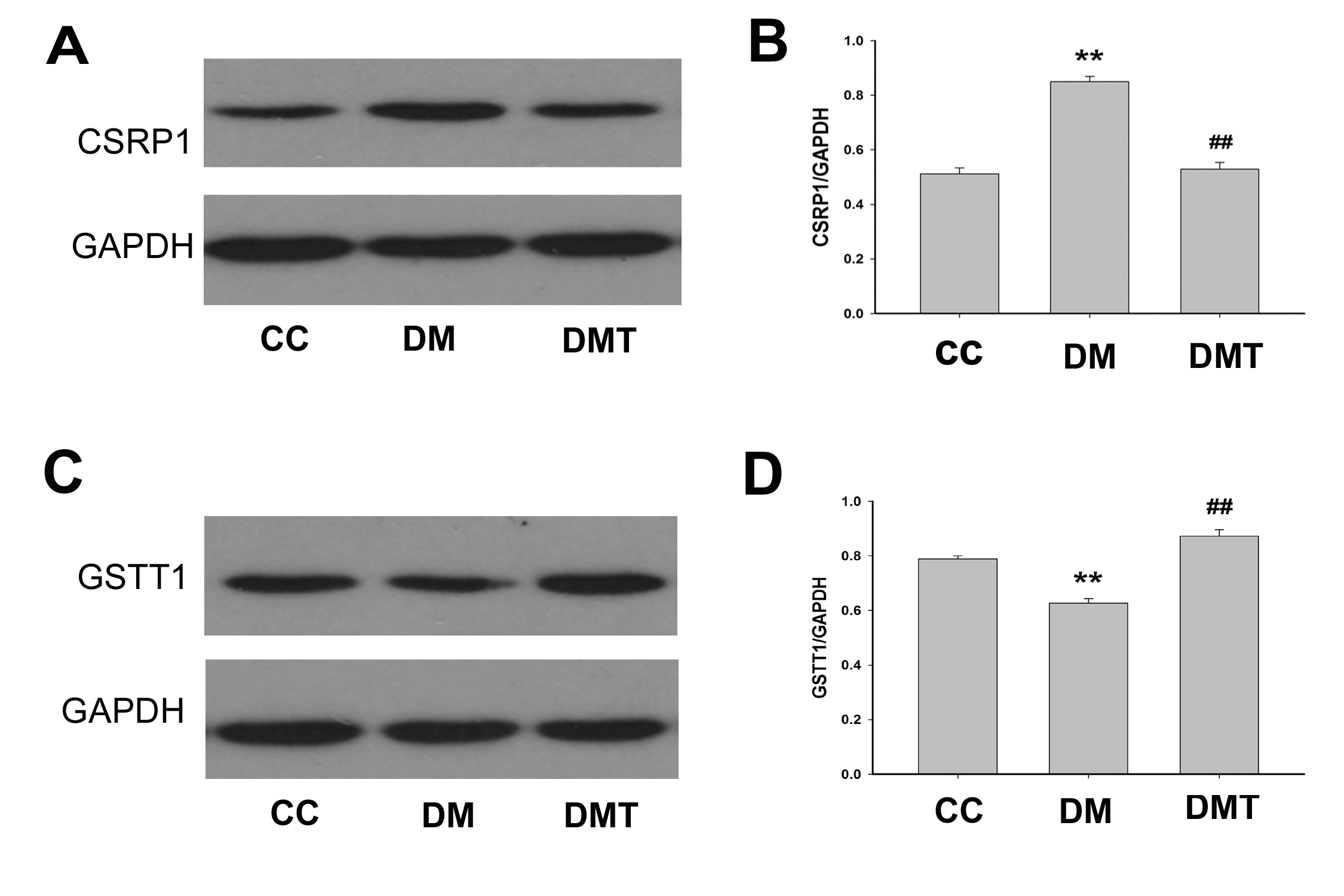

Supplement: Figure S1 — Western blotting validation of iTRAQ data on other two proteins: CSRP1 and GSTT1. GAPDH was used as the loading control. CC: control db/m group; DM: untreated db/db group; DMT: GSPB2 treated db/db group. **P<0.01 compared with CC group; ## P<0.01 compared with DM group. (TIF) [file pone.0052541.s001.tif]
