# Supplementary material for: Proteomic Analysis of Aorta and Protective Effects of Grape Seed Procyanidin B2 in db/db Mice Reveal a Critical Role of Milk Fat Globule Epidermal Growth Factor-8 in Diabetic Arterial Damage
Source: PLoS One. 2012 Dec 21;7(12):e52541. doi: 10.1371/journal.pone.0052541 (PMC3528673; doi:10.1371/journal.pone.0052541)
Supplement: Materials and Methods S1 — (DOCX) [file pone.0052541.s002.docx]

**Materials and Methods S1**

**Proteomic Analysis**

*Sample Preparation*

About 50mg Aortic tissue from each of four mice per group was pooled and homogenized in the presence of liquid nitrogen, and then lysed with 500μl STD buffer (4% SDS, 100mM DTT, 150mM TrisHCl pH 8.0). After 5 min incubation in boiling water, the suspensions were sonicated using ultrasonic cell crusher for 6 min (10 times, 80w, 10s each time with 15s interval).Then the mixture was incubated at 100°C for 5min. The crude extract was clarified by centrifugation at 14000g for 20 min.

*Trypsin Digestion*

Filer-aided sample preparation (FASP) method allows gel-free processing of biological samples solubilized with detergents for proteomic analysis by mass spectrometry. In FASP detergents are removed by ultrafiltration, and after protein digestion, peptides are separated from undigested material [1]. 120ug of proteins for each sample were incorporated into 30μl STD buffer, incubated at boiling water for 5min, cooling to room temperature, diluted with 200μl UA buffer (8 M Urea, 150 mM TrisHCl, pH8.0) and transferred to 30 kd ultrafiltration. The samples were centrifuged at 14000g for 15 min and 200μl UA buffer was added. The samples were centrifuged for 15 min at the same conditions. Then 100 μl 0.05 M iodoacetamide in UA buffer was added and the samples were incubated for 20 min in darkness. After 10 min centrifugation at the above conditions, the filters were washed three times with 100 μl UA buffer. Then 100 μl DS buffer (50 mM triethylammoniumbicarbonate at pH 8.5) were added to the filters and the samples were centrifuged for 10 min at the same conditions as before. This step was repeated twice. Finally, 2 μg trypsin (Promega) in 40 μl DS buffer were added to each filter. The samples were incubated overnight at 37°C or 25°C, respectively. The resulting peptides were collected by centrifugation. The filters were rinsed by 40 μl 10×DS buffer.

*iTRAQ Labeling and SCX Separation*

Concentration of the peptides can be estimated by UV spectrometer assuming that 0.1% solution of vertebrate proteins has at 280 nm an extinction of 1.1 absorbance units. About 60ug peptides of each group were labeled with iTRAQ reagents (114 for the peptides of CC group, 115 for the peptides of DMT group, and 117 for the peptides of DM group respectively) following the manufacturer’s instructions (Applied Biosystems).

The labeled samples were dried out and then diluted with 20 folds of Cation Exchange binding Buffer (10mM KH_2_PO4 in 25% acetonitrile at PH 3.0). Strong Cation Exchange (SCX) chromatography was performed to separate the labeled samples into 10 fractions by polysulfoethyl A column (4.6x100mm 5µ, 200Å, PolyLC). A suitable gradient elution was applied to separate peptides at a flow rate of 1ml/min with Elution buffer (10mM KH2PO4, 500mM KCl in 25% acetonitrile at PH 3.0). Eluted peptides were collected and desalted by an offline fraction collector and C18 cartridges (Sigma).

**References S1**

Wisniewski JR, Zielinska DF, Mann M (2011) Comparison of ultrafiltration units for proteomic and N-glycoproteomic analysis by the filter-aided sample preparation method. Anal Biochem 410: 307-309.
