# Supplementary material for: Proteomic Analysis of Aorta and Protective Effects of Grape Seed Procyanidin B2 in db/db Mice Reveal a Critical Role of Milk Fat Globule Epidermal Growth Factor-8 in Diabetic Arterial Damage
Source: PLoS One. 2012 Dec 21;7(12):e52541. doi: 10.1371/journal.pone.0052541 (PMC3528673; doi:10.1371/journal.pone.0052541)
Supplement: Table S1 — Differentially expressed proteins as reversed by GSPB2 identified by iTRAQ (DOC) [file pone.0052541.s003.doc]

**Table S1**

**Table S1. Differentially expressed proteins as reversed by GSPB2 identified by iTRAQ**

| Protein name | Accession no. | Molecular weight (Da) | PI | Protein expression ratio (DM/C) | Protein expression ratio (DMT/DM) | Function |
| --- | --- | --- | --- | --- | --- | --- |
| Milk fat globule-EGF factor 8 | IPI00788387.1 | 51240.91 | 6.10 | 2.41 | 0.59 | Cell adhesion, phagocytosis |
| Oxysterol-binding protein | IPI00831671.1 | 96552.79 | 6.51 | 8.93 | 0.35 | Lipid transport |
| DnaJ (Hsp40) homolog, subfamily C, member 13 | IPI00666885.3 | 254441.25 | 6.32 | 2.78 | 0.61 | Heat shock protein binding |
| Fructose-bisphosphate aldolase B | IPI00127206.6 | 39506.96 | 8.52 | 5.73 | 0.33 | Glycolysis |
| Isoform 3 of Latent-transforming growth factor beta-binding protein 1 | IPI00830551.1 | 147418.65 | 4.85 | 2.17 | 0.66 | Transforming growth factor beta receptor signaling pathway |
| Isoform 2 of PRKC apoptosis WT1 regulator protein | IPI00816941.1 | 30944.27 | 8.77 | 3.56 | 0.41 | Apoptosis |
| Steryl-sulfatase | IPI00118038.1 | 66591.04 | 8.83 | 2.63 | 0.54 | Lipid metabolism |
| Thyroid hormone-inducible hepatic protein | IPI00123449.1 | 17093.36 | 4.90 | 3.31 | 0.43 | Lipid synthesis |
| Isoform 2 of Inverted formin-2 | IPI00762596.3 | 137496.64 | 5.19 | 3.14 | 0.42 | Actin cytoskeleton organization |
| Cysteine and glycine-rich protein 2 | IPI00470178.7 | 20925.76 | 8.95 | 2.13 | 0.59 | Promote smooth muscle cell proliferation and dedifferentiation |
| Activator of 90 kDa heat shock protein ATPase homolog 1 | IPI00153740.1 | 38117.05 | 5.41 | 1.87 | 0.64 | Response to stress |
| Four and a half LIM domains 3 | IPI00828338.1 | 31794.7 | 5.80 | 2.51 | 0.44 | Actin cytoskeleton organization |
| Cysteine and glycine-rich protein 1 | IPI00123891.3 | 20583.34 | 8.90 | 1.79 | 0.60 | Actin cytoskeleton organization |
| Ig mu chain C region secreted form | IPI00468055.3 | 49943.89 | 6.60 | 2.20 | 0.48 | Activation of MAPK activity |
| Adenylyl cyclase-associated protein | IPI00882316.1 | 39877.39 | 5.43 | 11.19 | 0.53 | Cell morphogenesis, cytoskeleton organization |
| Lysozyme C-2 | IPI00107952.2 | 18312.86 | 9.11 | 10.64 | 0.53 | Bacteriolytic enzyme |
| Importin 9 | IPI00673797.2 | 115951.08 | 4.72 | 5.33 | 0.58 | Ran GTPase binding |
| Arf-GAP with SH3 domain, ANK repeat and PH domain-containing protein 2 isoform b | IPI00848371.1 | 106405.45 | 6.11 | 4.38 | 0.65 | GTPase activation |
| Lamin-B receptor | IPI00331173.8 | 71440.42 | 9.43 | 7.05 | 0.35 | DNA binding |
| GTP-binding protein 1 | IPI00761677.1 | 72300.66 | 8.57 | 4.09 | 0.59 | GTPase activation |
| Protein phosphatase 1 regulatory subunit 12C | IPI00669561.2 | 84685.71 | 5.73 | 5.89 | 0.40 | Regulates myosin phosphatase activity |
| Adenine phosphoribosyltransferase | IPI00625950.2 | 19724.03 | 6.31 | 3.92 | 0.58 | Adenine salvage |
| Alpha-1-antitrypsin 1-4 | IPI00123924.1 | 45974.61 | 5.32 | 3.23 | 0.65 | Inhibitor of serine proteases |
| Importin-7 | IPI00331444.7 | 119486.71 | 4.70 | 4.33 | 0.46 | Protein transport |
| Dynein light chain roadblock-type 1 | IPI00750671.1 | 11927.74 | 5.83 | 3.98 | 0.49 |  |
| Isoform 2 of Low molecular weight phosphotyrosine protein phosphatase | IPI00828470.1 | 17941.45 | 6.57 | 2.63 | 0.64 | Acid phosphatase activity |
| Protein S100-A4 | IPI00124096.1 | 11721.44 | 5.23 | 2.66 | 0.63 | Calcium ion binding |
| Serine carboxypeptidase 1, isoform CRA_b | IPI00895390.1 | 50958.64 | 5.56 | 2.72 | 0.62 | Proteolysis |
| Calsequestrin-1 | IPI00466657.4 | 46378.25 | 3.96 | 14.72 | 0.11 | Regulation of skeletal muscle contraction by regulation of release of sequestered calcium ion |
| Septin 8 | IPI00928138.1 | 50895.75 | 5.74 | 2.90 | 0.57 | GTP binding |
| Signal peptidase complex subunit 2 | IPI00228236.6 | 24977.65 | 8.75 | 2.49 | 0.64 | Peptidase activity |
| Plasminogen | IPI00322936.1 | 90781.95 | 6.21 | 2.65 | 0.60 | Tissue remodeling |
| NADH-ubiquinone oxidoreductase chain 4 | IPI00116843.1 | 51881.71 | 9.42 | 4.16 | 0.37 | Mitochondrial electron transport |
| Endophilin-A2 | IPI00312752.3 | 41518.31 | 5.53 | 2.67 | 0.57 | Endocytosis |
| CTP synthase 1 | IPI00111959.2 | 66682.55 | 6.14 | 2.25 | 0.66 | Pyrimidine biosynthesis |
| D-3-phosphoglycerate dehydrogenase | IPI00225961.5 | 56585.39 | 6.12 | 5.08 | 0.29 | Amino-acid biosynthesis |
| Eukaryotic translation initiation factor 5A-1 | IPI00648714.1 | 23158.35 | 4.74 | 2.21 | 0.65 | Protein biosynthesis |
| Von Willebrand factor A domain-containing protein 5A | IPI00221817.5 | 87142.79 | 6.15 | 2.53 | 0.56 | Tumor suppressor |
| RuvB-like 2 | IPI00123557.3 | 51112.54 | 5.49 | 2.23 | 0.64 | DNA recombination |
| Similar to thrombospondin 1 | IPI00669935.3 | 138599.13 | 4.85 | 2.55 | 0.55 |  |
| Exocyst complex component 3 | IPI00454030.3 | 86454.73 | 5.86 | 2.17 | 0.65 | Exocytosis |
| Ras-related protein Rab-5A | IPI00132410.1 | 23598.61 | 8.32 | 2.48 | 0.56 | Protein transport |
| Hsp90 co-chaperone Cdc37 | IPI00117087.1 | 44593.53 | 5.24 | 2.82 | 0.49 | Binds to numerous kinases and promotes their interaction with the Hsp90 complex |
| Filamin-binding LIM protein 1 | IPI00776395.1 | 41026.22 | 6.09 | 1.97 | 0.66 | Zinc ion binding |
| Nucleosome assembly protein 1-like 1 isoform 1 | IPI00929813.1 | 48543.12 | 4.36 | 2.21 | 0.58 | Nucleosome assembly |
| Dynein light chain 2, cytoplasmic | IPI00132734.1 | 10349.81 | 6.81 | 2.44 | 0.51 | Intracellular transport |
| Multiple coagulation factor deficiency protein 2 homolog | IPI00170316.1 | 16168.08 | 4.55 | 2.82 | 0.44 | Protein transport |
| High-mobility group (nonhistone chromosomal) protein 1-like 1 | IPI00875792.1 | 25027.05 | 5.92 | 2.27 | 0.54 | DNA binding |
| Abl-interactor 1 | IPI00919052.1 | 49138.87 | 7.16 | 2.60 | 0.47 | Protein tyrosine kinase activator activity |
| Ppp2r5e protein | IPI00654180.1 | 50830.43 | 8.64 | 2.02 | 0.60 | Signal transduction |
| Lamina-associated polypeptide 1B | IPI00762273.1 | 66780.5 | 6.74 | 2.11 | 0.57 | Protein binding |
| Fam129b 28 kDa protein | IPI00753430.1 | 28341.47 | 6.12 | 1.96 | 0.60 |  |
| Isoform 2 of Poly(ADP-ribose) glycohydrolase ARH3 | IPI00828376.1 | 31043.07 | 4.94 | 1.93 | 0.61 | Hydrolase |
| Eukaryotic translation initiation factor 3 subunit A | IPI00129276.2 | 161937.41 | 6.38 | 2.13 | 0.55 | Protein biosynthesis |
| Apex1 32 kDa protein | IPI00928109.1 | 32318.93 | 8.79 | 2.31 | 0.51 |  |
| Translationally-controlled tumor protein | IPI00129685.2 | 20249.19 | 4.84 | 2.43 | 0.48 | Calcium binding |
| Ig gamma-1 chain C region secreted form | IPI00830215.1 | 35704.47 | 7.23 | 1.85 | 0.63 | Phagocytosis, engulfment |
| Serine/threonine-protein phosphatase 2A activator | IPI00118723.3 | 36710.18 | 5.95 | 2.00 | 0.58 | ATP catabolic process |
| Calumenin | IPI00135186.1 | 37063.81 | 4.49 | 1.85 | 0.63 | Calcium ion binding |
| Annexin A1 | IPI00230395.5 | 38734.3 | 6.97 | 2.42 | 0.48 | Calcium ion binding, regulation of cell proliferation |
| 60S ribosomal protein L22 | IPI00222546.5 | 14758.89 | 9.21 | 1.97 | 0.59 | Alpha-beta T cell differentiation |
| Myeloid-associated differentiation marker | IPI00132938.2 | 35284.6 | 8.69 | 2.77 | 0.42 |  |
| Leucine-rich alpha-2-glycoprotein 1 | IPI00129250.1 | 37431.04 | 6.14 | 2.52 | 0.45 | Brown fat cell differentiation |
| Trafficking protein particle complex subunit 4 | IPI00112785.1 | 24385.12 | 5.84 | 1.99 | 0.57 | Dendrite development |
| Myosin, light polypeptide 1 | IPI00895557.1 | 16124.37 | 4.86 | 2.79 | 0.41 | Calcium ion binding |
| Cald1 Protein | IPI00757635.1 | 89361.07 | 5.49 | 1.70 | 0.66 |  |
| Rpl7 Protein | IPI00775948.1 | 32416.71 | 11.13 | 1.78 | 0.63 |  |
| Programmed cell death protein 6 | IPI00121736.1 | 21867.48 | 5.16 | 1.76 | 0.63 | Apoptosis |
| Four and a half LIM domains protein 1 isoform 2 | IPI00776352.1 | 33563.5 | 8.84 | 1.84 | 0.60 | Zinc ion binding |
| Microtubule-associated protein 1S | IPI00223621.1 | 102943.71 | 6.58 | 1.83 | 0.61 | Apoptosis |
| Inositol polyphosphate-5-phosphatase A isoform a | IPI00856931.1 | 47622.28 | 6.44 | 1.72 | 0.63 | Inositol or phosphatidylinositol phosphatase activity |
| Isoform 1 of Protein phosphatase 1 regulatory subunit 12B | IPI00876214.1 | 109049.69 | 5.58 | 2.45 | 0.44 | Regulates myosin phosphatase activity |
| Alpha-2-macroglobulin | IPI00624663.3 | 167282.41 | 6.38 | 1.74 | 0.62 | Serine-type endopeptidase inhibitor activity |
| Pigment epithelium-derived factor | IPI00331088.1 | 46234.13 | 6.48 | 2.12 | 0.51 | Negative regulation of angiogenesis |
| Ribosomal protein L18A family member | IPI00880213.1 | 22377.21 | 10.72 | 2.23 | 0.48 |  |
| Metaxin-1 isoform 2 | IPI00938432.1 | 51213.91 | 9.62 | 1.89 | 0.56 |  |
| Matrin-3 | IPI00453826.2 | 94630.58 | 5.87 | 1.70 | 0.62 | RNA binding |
| Phosphoribosyl pyrophosphate synthetase-associated protein 1 | IPI00755389.2 | 42467.65 | 8.62 | 1.97 | 0.54 | Nucleotide biosynthesis |
| Alpha-actinin-4 | IPI00118899.1 | 288825.17 | 5.71 | 1.62 | 0.65 | Protein transport |
| Hexaprenyldihydroxybenzoate methyltransferase, mitochondrial | IPI00467124.2 | 40956.59 | 8.08 | 2.05 | 0.51 | Ubiquinone biosynthesis |
| Isoform 2 of Tropomyosin beta chain | IPI00874728.1 | 32957.91 | 4.63 | 1.67 | 0.63 | Muscle contraction |
| Isoleucyl-tRNA synthetase, mitochondrial | IPI00453499.3 | 112804.36 | 6.37 | 2.81 | 0.37 | Protein biosynthesis |
| Isoform 1 of Tropomyosin beta chain | IPI00123319.1 | 32957.91 | 4.63 | 1.73 | 0.60 | Muscle contraction |
| Putative ATP-dependent Clp protease proteolytic subunit, mitochondrial | IPI00133270.1 | 29800.55 | 7.05 | 2.24 | 0.46 | Proteolysis |
| Proteasome subunit alpha type-4 | IPI00277001.4 | 29470.78 | 7.58 | 1.55 | 0.66 | Ubiquitin-dependent protein catabolic process |
| microtubule-associated protein 4 | IPI00848818.1 | 117442.17 | 4.90 | 1.55 | 0.66 | Negative regulation of microtubule depolymerization |
| Ras-related protein Rab-7a | IPI00408892.2 | 23489.72 | 6.40 | 1.66 | 0.62 | Protein transport |
| Isoform 2 of Sorcin | IPI00556765.1 | 20295.85 | 5.11 | 1.72 | 0.60 | Calcium ion transport |
| Myosin regulatory light chain 12B | IPI00132705.1 | 19854.22 | 4.80 | 1.72 | 0.60 | Calcium ion transport |
| Isoform 2 of Protein NDRG2 | IPI00759847.1 | 39280.55 | 5.4 | 1.71 | 0.60 | Differentiation neurogenesis |
| Ras-related protein Rab-11B | IPI00135869.3 | 24489.49 | 5.64 | 1.82 | 0.56 | Protein transport |
| CD99 antigen | IPI00187438.1 | 16782.58 | 5.14 | 1.73 | 0.59 | Protein binding |
| V-type proton ATPase subunit B, brain isoform | IPI00119113.3 | 56550.91 | 5.57 | 2.25 | 0.45 | ATP hydrolysis coupled proton transport |
| Isoform 1 of Filamin-A | IPI00131138.10 | 281193.28 | 5.68 | 1.61 | 0.63 | Actin crosslink formation |
| dipeptidyl-peptidase 3 | IPI00938439.1 | 82898.06 | 5.26 | 1.86 | 0.55 | Proteolysis |
| Adenylosuccinate lyase | IPI00308217.1 | 54808.17 | 6.90 | 1.58 | 0.64 | Purine biosynthesis |
| Calcium/calmodulin-dependent protein kinase type II subunit beta | IPI00875723.1 | 55960.72 | 6.73 | 1.58 | 0.64 | Induction of apoptosis in response to chemical stimulus |
| Lupus La protein homolog | IPI00134300.1 | 47756.4 | 9.77 | 1.58 | 0.64 | RNA processing |
| Hypothetical protein isoform 1 | IPI00677102.1 | 14334.62 | 10.52 | 1.87 | 0.59 |  |
| Similar to IKK interacting protein | IPI00850164.1 | 46079.28 | 4.98 | 2.02 | 0.61 |  |
| Uncharacterized protein Cald1 | IPI00775950.1 | 89361.07 | 5.49 | 1.53 | 0.66 | Muscle contraction |
| Putative uncharacterized protein Sfxn3 | IPI00653231.1 | 30951.62 | 9.18 | 1.60 | 0.64 | Cation transmembrane transporter activity |
| Uncharacterized protein Rcn3 | IPI00895423.1 | 31843.11 | 4.75 | 2.55 | 0.42 | Calcium ion binding |
| Putative uncharacterized protein Gm9892 | IPI00480398.1 | 38120.17 | 5.72 | 2.28 | 0.47 |  |
| Putative uncharacterized protein Ncstn | IPI00928374.1 | 78492.08 | 5.75 | 1.72 | 0.65 | Membrane protein ectodomain proteolysis |
| Putative uncharacterized protein Plcb4 | IPI00625848.2 | 134527.78 | 6.47 | 2.26 | 0.52 | Lipid metabolic process |
| Putative uncharacterized protein Gm2178 | IPI00848921.1 | 13405 | 10.31 | 1.87 | 0.63 | Translation |
| Putative uncharacterized protein Batla | IPI00652895.1 | 35986.72 | 6.52 | 1.98 | 0.62 | ATP binding |
| Putative uncharacterized protein Myo6 | IPI00776187.1 | 144773.87 | 8.75 | 2.69 | 0.49 | Proteolysis |
| Putative uncharacterized protein Ctsd | IPI00404551.1 | 48373.86 | 6.85 | 2.22 | 0.60 | Proteolysis |
| Uncharacterized protein Wdfy1 | IPI00831243.1 | 20539.26 | 6.53 | 6.02 | 0.64 |  |
| Uncharacterized protein Flt3l | IPI00919200.1 | 48342.56 | 10.25 | 2.29 | 0.63 | Ribonucleoprotein |
| Putative uncharacterized protein Vapb | IPI00654073.1 | 22737.25 | 7.86 | 5.08 | 0.37 | Structural molecule activity |
| Putative uncharacterized protein Lrrc59 | IPI00653744.1 | 20408.49 | 6.18 | 1.97 | 0.57 |  |
| Isoform 1 of Apoptosis-inducing factor 2 | IPI00225407.1 | 40634.88 | 9.09 | 0.27 | 2.61 | Oxidation-reduction process |
| Glutathione S-transferase theta-1 | IPI00881469.1 | 27374.28 | 6.79 | 0.63 | 1.83 | Glutathione metabolic process |
| NEDD8 ultimate buster 1 | IPI00108775.3 | 70306.71 | 5.64 | 0.07 | 4.25 | Protein ubiquitination |
| Isochorismatase domain-containing protein 2A, mitochondrial | IPI00757372.2 | 22417.37 | 8.25 | 0.34 | 1.51 | Catalytic activity |
| SUMO-activating enzyme subunit 2 | IPI00130173.1 | 70568.9 | 5.09 | 0.27 | 1.96 | UbI conjugation pathway |
| Similar to MRC OX-2 antigen homolog | IPI00853751.1 | 10956.24 | 6.96 | 0.45 | 1.72 |  |
| Isoform 10 of Myelin basic protein | IPI00223593.1 | 20814.15 | 10.86 | 0.29 | 2.78 | Myelination |
| Mitochondrial ribosomal protein S23 | IPI00882252.1 | 18283.79 | 7.78 | 0.54 | 1.50 | Translation |
| Myosin-IXa | IPI00928546.1 | 301836.93 | 9.05 | 0.28 | 2.96 | Intracellular signal transduction |
| Isoform 1 of Methylglutaconyl-CoA hydratase, mitochondrial | IPI00124900.1 | 33395.05 | 9.56 | 0.51 | 1.68 | Branched-chain amino acid catabolism |
| Isoform Long of Estradiol 17-beta-dehydrogenase 8 | IPI00230550.2 | 28396.34 | 7.70 | 0.39 | 2.26 | Fatty acid biosynthesis |
| Collagen alpha-1(X) chain | IPI00109296.1 | 66775.63 | 9.80 | 0.43 | 2.23 | A product of hyperthrophic chondrotocytes and has been localized to presumptive mineralization zones of hyaline cartilage |
| Poly(rC)-binding protein 1 | IPI00128904.1 | 37497.77 | 6.66 | 0.49 | 1.97 | mRNA processing |
| Mitochondrial fission 1 protein isoform 2 | IPI00857192.1 | 16264.78 | 9.28 | 0.64 | 1.54 | Apoptosis |
| Drebrin isoform 2 | IPI00956958.1 | 72545.09 | 4.39 | 0.50 | 2.05 | Differentiation neurogenesis |
| Isoform 1 of Microtubule-actin cross-linking factor 1 | IPI00890208.1 | 601774.9 | 5.34 | 0.64 | 1.70 | Wnt receptor signaling pathway |
| cGMP-specific 3',5'-cyclic phosphodiesterase | IPI00947583.1 | 98407.54 | 5.70 | 0.16 | 7.18 | cGMP catabolic process |
| Putative uncharacterized protein Cops6 | IPI00830393.1 | 33591.61 | 5.65 | 0.59 | 1.79 | Protein binding |
| Putative uncharacterized protein Hnrnph3 | IPI00885560.1 | 35181.64 | 6.36 | 0.31 | 2.91 | Nucleic acid binding |
| Putative uncharacterized protein Tst | IPI00762128.2 | 33480 | 7.71 | 0.33 | 1.68 | Sulfate transport |
| Uncharacterized protein KIAA0564 homolog | IPI00929874.1 | 116185.15 | 6.61 | 0.42 | 1.75 | ATP binding |
| Uncharacterized protein Fmr1 | IPI00776271.1 | 70811.24 | 7.28 | 0.46 | 2.00 | RNA binding |
| Putative uncharacterized protein Pfkp | IPI00927975.1 | 85300.68 | 6.62 | 0.44 | 2.12 | Glycolysis |
| Putative uncharacterized protein Pigs | IPI00654122.1 | 61614.67 | 6.48 | 0.30 | 3.80 |  |
| Putative uncharacterized protein Pnp2 | IPI00875288.1 | 32284.18 | 6.10 | 0.54 | 2.20 | Nucleoside metabolic process |
